# Proteasome Inhibitors for the Treatment of Multiple Myeloma

**DOI:** 10.3390/cancers12020265

**Published:** 2020-01-22

**Authors:** Shigeki Ito

**Affiliations:** Hematology & Oncology, Department of Internal Medicine, Iwate Medical University School of Medicine, Yahaba-cho 028-3695, Japan; shigei@iwate-med.ac.jp; Tel.: +81-19-613-7111

**Keywords:** multiple myeloma, proteasome inhibitors, bortezomib, carfilzomib, ixazomib

## Abstract

Use of proteasome inhibitors (PIs) has been the therapeutic backbone of myeloma treatment over the past decade. Many PIs are being developed and evaluated in the preclinical and clinical setting. The first-in-class PI, bortezomib, was approved by the US food and drug administration in 2003. Carfilzomib is a next-generation PI, which selectively and irreversibly inhibits proteasome enzymatic activities in a dose-dependent manner. Ixazomib was the first oral PI to be developed and has a robust efficacy and favorable safety profile in patients with multiple myeloma. These PIs, together with other agents, including alkylators, immunomodulatory drugs, and monoclonal antibodies, have been incorporated into several regimens. This review summarizes the biological effects and the results of clinical trials investigating PI-based combination regimens and novel investigational inhibitors and discusses the future perspective in the treatment of multiple myeloma.

## 1. Introduction

Multiple myeloma (MM) remains an incurable disease. Over the last ten years, the availability of new drugs, such as the proteasome inhibitors (PIs), the immunomodulatory drugs (IMiDs), the monoclonal antibodies (MoAbs), and the histone deacetylase inhibitors, have greatly advanced the treatment and improved the survival of patients with MM [1,2,3]. Proteasome inhibition has emerged as a crucial therapeutic strategy in the treatment of MM. The first-in-class PI, bortezomib, was approved by the US food and drug administration (FDA) in 2003; it has contributed to the improvement in survival of MM patients [4]. Bortezomib is currently widely used not only for the patients with newly diagnosed but also relapsed and/or refractory MM. Recently, several second-generation PIs, including carfilzomib and ixazomib, have been introduced into clinical use. Carfilzomib is a peptide epoxyketone that binds irreversibly to the β5 subunit of the constitutive proteasome and inhibits the chymotrypsin-like activity [5]. Carfilzomib was approved by the US FDA in 2012; it is indicated as a single agent, or in combination with dexamethasone (Kd) or with lenalidomide plus dexamethasone (KRd) for the treatment of patients with relapsed and/or refractory MM (RRMM). Ixazomib is an orally bioavailable PI that preferentially binds to the β5 subunit of the 20S proteasome and inhibits the chymotrypsin-like activity. Ixazomib was approved by the US FDA in 2015; it is indicated in combination with lenalidomide and dexamethasone for the treatment of patients with RRMM. This review provides an overview of the biological effects and pivotal clinical trials of these PI-combination regimens, and discusses the future outlook with regards to the treatment of MM.

## 2. Proteasome Inhibitors

PIs have different chemical structures, and distinct pharmacokinetics and pharmacodynamics characteristics (Table 1) [6,7]. New PIs, including marizomib (NPI-0052), oprozomib (ONX0912), and delanzomib (CEP-18770), are being investigated.

### Mechanism of Action of PIs

The ubiquitin proteasome pathway (UPP) is the essential mechanism for protein catabolism in the nucleus and cytoplasm of eukaryotic cells. The proteasome is a large multi-protein complex consisting of multicatalytic proteases and is responsible for degrading or processing intracellular proteins. The 20S proteasome is created by four stacked rings which contain 28 protein subunits. Two of these rings consist of seven α-subunits and the other two consist of seven β-subunits. Three (β1, β2, and β5) of the seven β-subunits have proteasome enzymatic activities that have been characterized as chymotrypsin-like [8]. In MM cells, large amounts of monoclonal proteins are produced and secreted. By inhibiting proteasome function, misfolded or unfolded proteins are accumulated in the endoplasmic reticulum (ER), which is known as ER stress. ER stress leads to activate pro and antiproliferative signals, disrupts cell cycle regulation, activates apoptotic pathways, and ultimately, results in cell death [9,10]. A recent report demonstrated that co-inhibition of β1 or β2 with β5 activity achieves meaningful functional proteasome inhibition and cytotoxicity in MM cells [11]. Interestingly, only high-dose carfilzomib from the PIs that are currently available has been shown to provide β2/β5 co-inhibition and to have a more potent cytotoxic effect in MM cells. These findings raise the possibility that high-dose carfilzomib might be effective in other PI-resistant MM cells.

**Figure 1:**
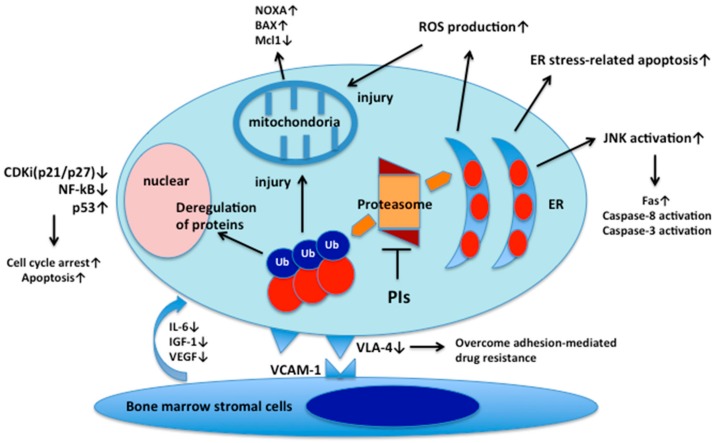
Molecular effects of proteasome inhibition on multiple myeloma cells.

Besides the ER stress-related apoptosis by proteasome inhibition as described above, there are a number of molecular effects of proteasome inhibition on MM cells (Figure 1). The classical mechanism of action of bortezomib on MM cells was an inhibitory effect on the transcription factor nuclear factor-κB (NF-κB) pathway activation, which is essential for myelomagenesis [12,13]. NF-κB induces several growth and angiogenesis factors including interleukin-6 (IL-6) and vascular endothelial growth factor (VEGF), cell cycle regulators including c-Myc and cyclin D1 and enhances the adherence of MM cells to stromal cells. PIs suppress NF-κB activity through stabilizing the inhibitory molecule IκB [14]. However, a recent study has demonstrated that bortezomib promotes constitutive NF-κB activity in MM cells, suggesting that the effects of bortezomib on the NF-κB pathway may be different among cell types and dominant types of canonical or non-canonical NF-κB pathways in MM cells [15,16]. Other putative mechanism of cellular toxicity induced by PIs is direct induction of apoptosis via c-Jun NH2-terminal kinase (JNK) and p53 [17,18]. The JNK activation leads to apoptosis through the upregulation of Fas and activation of caspase-8 and caspase-3. Accumulation and phosphorylation p53 by proteasome inhibition induces pro-apoptotic proteins including NADPH oxidase activator (NOXA) and Bcl-2-associated X protein (Bax), resulting in apoptosis via mitochondrial dysfunction, such as cytochrome-c release and membrane potential dysregulation. Bortezomib also decreases the expression of several cell adhesion molecules, such as very late antigen (VLA)-4, leading to resensitizing adhesion-mediated drug resistance of MM cells [19].

Proteasome inhibitors (PIs) induces the accumulation of unfolded and misfolded proteins, leading to apoptosis and cell death through ER stress, reactive oxygen species production, JNK and p53 activation, cyclin-dependent kinase inhibitors, and pro-apoptotic proteins induction. Bortezomib inhibits production of cytokines such as IL-6, insulin-like growth factor-1 (IGF-1), and VEGF in bone marrow stromal cells. Bortezomib also inhibits VLA-4 expression, resulting in overcoming cell adhesion-mediated drug resistance.

## 3. Bortezomib

Bortezomib is a boronic acid dipeptide that reversibly binds to the chymotrypsin- and caspase-like active sites and inhibits these enzyme activities [6,20]. Bortezomib has been shown to directly lead to apoptosis of MM cells, inhibit the NF-κB activation both in cells and tumor microenvironment, and inhibit adherence of myeloma cells to bone marrow stromal cells [14,21,22].

### 3.1. Bortezomib-Based Combinations for Transplant-Eligible Patients

Selected bortezomib-containing regimens for initial therapy in transplant-eligible patients with MM are shown in Table 2.

#### 3.1.1. Bortezomib and Dexamethasone (BD)

Several phase II studies evaluated bortezomib plus dexamethasone (BD) induction. The response rates were 66% to 90%, including 15% to 21% with complete response (CR) and 31.5% to 70% with a very good partial response (VGPR) or better [31,32,33]. Based on these promising results, the intergroupe francophone du Myélome (IFM) 2005-01 phase III study was conducted; it compared BD with vincristine, doxorubicin, and dexamethasone (VAD) as induction before autologous stem cell transplantation (ASCT) in previously untreated patients [23]. The response rates after induction were significantly higher in the BD group than in the VAD group (78.5% vs. 62.8%). After first ASCT, CR/near CR (nCR) and VGPR or better rates remained significantly higher in the BD group than in the VAD group (CR/nCR 35% vs. 18.4%, VGPR or better 54.3% vs. 37.2%, respectively). There was a trend for improved progression-free survival (PFS) in the BD group versus the VAD group (36.0 vs. 20.7 months, *p* = 0.06). However, three-year overall survival (OS) rates were not significantly different (81.4 vs. 77.4 months). This might be due to the influence of lenalidomide consolidation and maintenance for more than half the patients in each group. Although severe adverse events (AEs) were comparable between two groups, hematologic toxicity and treatment-related mortality were more frequently observed in the VAD group. On the other hand, grade 3 or 4 peripheral neuropathy (PN) during induction was more frequently observed in the BD group compared to the VAD group (9.2% vs. 2.5%).

#### 3.1.2. Bortezomib, Cyclophosphamide, and Dexamethasone (VCD)

Several studies have shown that a combination of bortezomib, cyclophosphamide, and dexamethasone (VCD) is an effective regimen, with favorable tolerability in relapsed and/or refractory MM [34,35,36,37]. The VCD regimen as induction therapy has also been shown to be effective, in several small studies, for patients with previously untreated MM [38,39,40]. The open-label, prospective, multicenter phase II, Deutsche studiengruppe multiples myeloma (DSMM) XI trial was conducted; this evaluated the efficacy and safety of VCD as induction therapy in 414 patients with newly diagnosed MM [41]. Patients received three 21-day cycles of VCD before ASCT. The overall response rate (ORR) was 85.4% and the rate of CR was 7.4%. The ORR after induction was similar between patients with or without high-risk cytogenetics (86.2% vs. 84.3%). At 55.5 months of a median follow-up, the median PFS and OS were 35.3 months and not reached, respectively. However, the median PFS was significantly shorter in patients with high-risk versus standard-risk cytogenetics (19.9 vs. 43.6 months, *p* < 0.0001), as well as median OS (54.7 vs. not reached, *p* = 0.0022). The most common grade 3 or higher AEs were leukopenia (31.4%) and thrombocytopenia (6.8%).

#### 3.1.3. Bortezomib, Thalidomide, and Dexamethasone (VTD)

Recently, the addition of a third agent to BD has been evaluated in phase II/III studies. According to the results, the efficacy of triplet regimens generally seemed better than doublet regimens. The GIMEMA Italian myeloma network reported the results of a randomized phase III study that compared bortezomib, thalidomide plus dexamethasone (VTD) with thalidomide plus dexamethasone (TD) as induction therapy before, and consolidation therapy after, double ASCT in previously untreated MM [25]. The primary endpoint, the CR or nCR rate after induction therapy was significantly higher in the VTD group versus the TD group (31% vs. 11%, *p* < 0.0001). After consolidation therapy, the CR or nCR rate was also significantly higher in the VTD group versus the TD group (62% vs. 45%, *p* = 0.0002). In addition, the median PFS was significantly longer in the VTD group versus the TD group (Hazard ration: HR 0.63, 95% 0.45–0.88, *p* = 0.0061). The estimated 3-year rate of PFS was 68% in the VTD group and 56% in the TD group (*p* = 0.0057). The 3-year OS was 86% in the VTD group and 84% in the TD group (*p* = 0.30). Grade 3 or 4 AEs were reported in a significantly higher number of patients on VTD (56%) than in those on TD (33%), with a higher incidence of PN in patients on VTD (10%) than in those on TD (5.2%). These results suggest that VTD induction therapy before ASCT significantly improves the rate of CR or nCR and PFS versus TD in transplant-eligible MM patients. In addition, the Spanish myeloma group reported the results of a randomized phase III trial comparing VTD versus TD versus vincristine, BCNU, melphalan, cyclophosphamide, plus prednisone, and vincristine, BCNU, doxorubicin, plus dexamethasone, and bortezomib (VBMCP/VBAD/B) in patients aged 65 years or younger with MM [26]. The primary endpoint was CR rate after induction therapy and ASCT. The CR rate was significantly higher in the VTD group than in the TD group (35% vs. 14%, *p* = 0.001) or in the VBMCP/VBAD/B group (35% vs. 21%, *p* = 0.01). The median PFS was significantly longer in the VTD group (56.2 vs. 28.2 vs. 35.5 months, *p* = 0.01). The CR rate after ASCT was higher in the VTD group than in the TD group (46% vs. 24%, *p* = 0.004) or in the VBMCP/VBAD/B group (46% vs. 38%, *p* = 0.1).

VCD has demonstrated a high response rate in prospective phase II and phase III clinical trials [24,39,40,41]. The IFM conducted a randomized trial of head-to-head comparison between VTD and VCD prior to ASCT in patients with untreated MM [27]. After four cycles, the rate of VGPR or better (primary endpoint) was significantly higher in the VTD group than in the VCD group (66.3% vs. 56.2%, *p* = 0.05). Hematologic toxicity was higher in the VCD group, with significantly increased rates of grade 3 or 4 anemia, thrombocytopenia, and neutropenia. Overall, these results show that the combination of a PI plus IMiD plus dexamethasone is the best option prior to intensive therapy and ASCT, although both VTD and VCD are active induction regimens.

#### 3.1.4. Bortezomib, Doxorubicin, and Dexamethasone (PAD)

The randomized phase III HOVON-65/GMMG-HD4 trial was conducted; this compared the efficacy and safety of VAD with bortezomib, doxorubicin plus dexamethasone (PAD) as pre-ASCT induction, followed by thalidomide and bortezomib maintenance [28]. The CR rate was significantly higher after PAD induction versus VAD (31% vs. 15%, *p* < 0.001) and bortezomib maintenance versus thalidomide (49% vs. 34%, *p* < 0.001). After a median follow-up of 41 months, significant longer PFS was observed in the PAD group than in the VAD group (35 vs. 28 months, HR 0.75, *p* = 0.002). Notably, OS was also superior after PAD plus bortezomib maintenance compared to VAD plus thalidomide maintenance. A benefit was also observed in patients with del(17p) (mPFS. 22 vs. 12 months, HR 0.47, *p* < 0.01; median OS, not reached vs. 24 months, HR 0.36, *p* = 0.003).

#### 3.1.5. Bortezomib, Lenalidomide, and Dexamethasone (VRD)

To prolong PFS and OS, one goal of frontline treatment is to maximize the depth of response. Although thalidomide and lenalidomide are both IMiDs, lenalidomide appears to be an ideal partner with bortezomib, rather than thalidomide, because the combination of bortezomib with thalidomide is limited by the occurrence of PN. A dose-escalation study demonstrated the promising efficacy and tolerability of bortezomib in combination with lenalidomide plus dexamethasone (VRD) as induction followed by ASCT and VRD maintenance [42]. On the basis of these studies, a randomized phase III IFM2009 trial was conducted; this evaluated the timing of ASCT in the era of triplet induction therapy with bortezomib and lenalidomide. Patients received induction therapy with three cycles of VRD and then consolidation with either five cycles of VRD or upfront ASCT followed by two additional cycles of VRD [29]. Lenalidomide as maintenance was given for one year in both groups. The median PFS was significantly longer in the upfront ASCT group than in the VRD-alone group (50 months vs. 36 months, HR 0.65, *p* < 0.001). The rate of CR was significantly higher in the ASCT group than in the VRD-alone group (59% vs. 48%, *p* = 0.03). Minimal residual disease (MRD) negativity was also higher in the ASCT group than in the VRD-alone group (79% vs. 65%, *p* < 0.001). However, OS at 4 years did not differ significantly between both groups (81% vs. 82%). These results indicated that upfront ASCT is still necessary and a standard of care in the era of VRd regimen. Recently, the phase III PETHEMA/GEM2012 study evaluated VRD for six cycles followed by ASCT conditioned with intravenous busulfan plus melphalan versus melphalan and post-transplant consolidation with two cycles of VRD [43]. The rates of CR and VGPR or better after induction therapy were 33.4% and 66.6%, respectively. A stringent CR (sCR) rate was 26.2%. In patients with high-risk cytogenetics, the rates of CR and VGPR or better were 34.8% and 70.7%, respectively. The CR rate after ASCT and consolidation were 44.1% and 50.2%. In addition, MRD negativity (median 3 × 10^−6^ sensitivity) increased from induction (28.8%) to transplant (42.1%) and consolidation (45.2%). The most common grade 3 or 4 hematological AEs during induction were neutropenia and thrombocytopenia (12.9% and 9.2%). Although grade 2 or higher PN during induction was observed in 17% of the patients, the frequency of grade 3 and 4 PN was low (3.7% and 0.2%). These results indicated that VRD induction and consolidation is highly effective and well-tolerated in previously untreated MM. VRD induction regimen is being most widely used at present.

#### 3.1.6. Daratumumab, Bortezomib, Thalidomide, and Dexamethasone (D-VTD)

VTD followed by ASCT is the standard treatment in Europe for transplant-eligible patients with previously untreated MM. A randomized, open-label, phase III CASSIOPEIA trial comparing CD38 monoclonal antibody, daratumumab plus VTD (D-VTD) with VTD alone was conducted [30]. Patients received four cycles of induction and two post-transplant consolidation cycles of D-VTD or VTD alone. The primary endpoint of part 1 was sCR assessed 100 days after ASCT. The rates of sCR and CR or better in the D-VTD group were 29% and 39%, and those in the VTD group were 20% and 26%. The MRD negativity (10^−5^ sensitivity threshold) was also higher in the D-VTD group than in the VTD group (64% vs. 44%, *p* < 0.0001), suggesting that addition of daratumumab to VTD leads to deeper response versus VTD alone. The median PFS was not reached in either group (HR 0.47, *p* < 0.0001). Grade 3 or 4 neutropenia were observed in 28% in the D-VTD group and 15% in the VTD group, respectively. This is the first study showing that the addition of daratumumab to standard of care has the significant clinical benefit in transplant-eligible patients with previously untreated MM.

### 3.2. Bortezomib-Based Combinations for Transplant-Ineligible Patients

Melphalan plus prednisone (MP) has historically been the most widely accepted treatment and considered as one of the standards of care in transplant-ineligible myeloma patients. Since novel agents have been developed, the treatment choices for newly diagnosed myeloma patients have dramatically changed. Selected bortezomib-containing regimens for initial therapy in transplant-ineligible patients with MM are shown in Table 3.

#### 3.2.1. Bortezomib, Melphalan, and Prednisone (VMP)

Bortezomib has a synergistic activity in vitro in combination with melphalan [48]. In the phase I or II studies of VMP, ORR and CR rate were 89% and 32%, respectively. Median time to progression (TTP) was 27 months and OS was 38 months [49,50]. Based on these promising results, the VISTA study was conducted; it compared VMP with MP for patients with previously untreated MM [44]. The primary endpoint, TTP in the VMP group, and in the control group was 24.0 and 16.6 months, respectively (hazard ratio (HR) = 0.48; *p* < 0.001). The ORR was also higher in the VMP group than in the control group (71% vs. 35%; *p* < 0.001), and the CR rates was also higher in the VMP group than in the control group (30% and 4%, *p* < 0.001). Notably, VMP was superior to MP in terms of OS (HR 0.61). An updated follow-up study confirmed a survival advantage for VMP [51]. Median OS was not reached in the VMP group and 43 months in the MP group, respectively. VMP regimen is now considered as one of the standards of care in transplant-ineligible patients with previously untreated MM.

#### 3.2.2. Daratumumab Plus VMP (D-VMP)

Daratumumab has a variety of mechanisms of action, including direct antitumor effect [52,53,54,55,56,57,58]. The ALCYONE trial has demonstrated the superiority of D-VMP versus VMP in terms of PFS. The 18-month PFS rate was 71.6% in the D-VMP group versus 50.2% in the control group (HR 0.50, *p* < 0.001) [45]. The ORR was significantly higher in the D-VMP group than in the VMP group (90.9% and 73.9%, *p* < 0.001). The rate of CR or better (including sCR) was also higher in the D-VMP group than in the control group (42.6% and 24.4%, *p* < 0.001). Notably, MRD negativity (10^−5^ sensitivity threshold) was higher in the D-VMP group than in the control group (22.3% and 6.2%, *p* < 0.001). Although the most common AEs of grade 3 or 4 were hematologic, the frequencies of neutropenia, thrombocytopenia, and anemia were comparable between both groups; neutropenia was observed in 39.9% in the D-VMP group and in 38.7% in the VMP group, thrombocytopenia in 34.4% and 37.6%, and anemia in 15.9% and 19.8%, respectively. The rate of grade 3 or 4 infections was higher in the D-VMP group than in the control group (23.1% and 14.7%). However, the treatment discontinuation rate due to infections was low (0.9% and 1.4%). Daratumumab-associated infusion-related reactions were observed in 27.7% of the patients. Thus, the addition of daratumumab on VMP did not increase overall toxicity except for infection. Daratumumab plus VMP is a new standard of care for patients with previously untreated MM who are ineligible for ASCT.

#### 3.2.3. Bortezomib, Lenalidomide, and Dexamethasone (VRd)

Lenalidomide and bortezomib have different, but synergistic, mechanisms of action [59]. Recently, the southwest oncology group (SWOG) study S0777 was reported; it compared VRd with Rd in newly diagnosed MM without intention for immediate ASCT [46]. After a median follow-up of 55 months, the median PFS was longer in the VRd group compared with the Rd group (43 months vs. 30 months, HR 0.712, *p* = 0.0037). The median OS was also longer in the VRd group compared with the Rd group (75 vs. 64 months; HR 0.709, *p* = 0.025). The rates of overall response were 82% in the VRd group and 72% in the Rd group. In addition, the rates of CR were 16% and 8%, respectively. Adverse events of grade 3 or higher were observed in 82% in the VRd group and 75% in the Rd group. The discontinuation rates due to AEs were 23% in the VRd group and 10% in the Rd group, respectively.

For elderly patients, the dosing schedule of VRd may be more challenging. Reduced dose and schedule of drugs may be more tolerable and maintain efficacy. Recently, a phase II study of modified VRd (VRd lite) was reported [47]. Fifty-three eligible patients were enrolled and 50 received at least one dose of therapy. The ORR and VGPR or better were obtained in 86% and 66% of patients, respectively. Median PFS was 35.1 months and the median OS was not reached at a median follow-up of 30 months. Notably, PN was observed in 31 (62%) patients with only one patient experiencing grade 3 symptoms. Overall, VRd lite seems likely to be a well-tolerated and highly effective regimen in transplant-ineligible MM patients and may represent the new standard of care.

## 4. Carfilzomib

Carfilzomib binds irreversibly to the β5 subunit of the constitutive proteasome and inhibits the chymotrypsin-like activity. The safety profile of this agent is quite different from other PIs. In particular, the agent is associated with cardiovascular AEs. Recently, Efentakis et al. demonstrated that cardiotoxicity by carfilzomib is associated with the autophagy pathway and upregulation of protein phosphatase (PP)-2A phosphatase activity but not the inhibition of the proteasome function [60]. Selected carfilzomib-containing regimens for relapsed and/or refractory MM patients are shown in Table 4.

### 4.1. Carfilzomib-Based Therapy in Relapsed and/or Refractory MM

#### 4.1.1. Carfilzomib and Dexamethasone (Kd)

A phase I study was conducted to evaluate the maximum-tolerated dose (MTD), pharmokinetics, and pharmacodynamics of carfilzomib administered as a 30-minute intravenous infusion [66]. Single-agent carfilzomib on days 1, 2, 8, 9, 15, and 16 of a 28-day cycle was given in thirty-three relapsed and refractory MM patients. The carfilzomib doses of days 1 and 2 on cycle 1 were 20 mg/m^2^, followed thereafter with doses escalated to 36, 45, 56, or 70 mg/m^2^. In addition, carfilzomib was combined with low-dose dexamethasone (40 mg/week). Dose-limiting toxicities were reported in two patients at 70 mg/m^2^, who had renal tubular necrosis and proteinuria. The MTD was established at 56 mg/m^2^. ORR was 50% in the 56 mg/m^2^ cohort.

In 2014, a single-arm/single-center phase II study of carfilzomib with or without low-dose dexamethasone in relapsed MM was published [67]. After two cycles of single-agent carfilzomib, 20 mg of dexamethasone could be added if the response was less than PR or if there was progressive disease. Forty-four patients were enrolled; all of these had had prior bortezomib and IMiDs and a median of five prior regimens. The ORR was 55% in 42 evaluable patients. Median duration of response (DOR), PFS, and OS were 11.7, 4.1, and 20.3 months, respectively. The most frequent grade 3 or 4 AEs were lymphopenia (43%), thrombocytopenia (32%), hypertension (25%), pneumonia (18%), and heart failure (11%). The discontinuation rate due to AEs was 16%.

Based on these promising results, the phase III ENDEAVOR trial comparing Kd with BD in 929 patients with RRMM was initiated [61]. The carfilzomib doses of days 1 and 2 on cycle 1 were 20 mg/m^2^, followed thereafter with doses escalated to 56 mg/m^2^. Bortezomib was administered twice-weekly subcutaneously or intravenously. Median PFS was significantly improved with the Kd group versus the BD group (18.7 vs. 9.4 months; HR 0.53, *p* < 0.0001). The ORR was higher in the Kd group than in the BD group (77% vs. 63%, *p* < 0.0001). The median time to response was 1.1 months in both groups. An updated analysis demonstrated that OS was also significantly improved with the carfilzomib group versus the bortezomib group (47.6 vs. 40.0 months, *p* = 0.010). Serious AEs were reported in 48% in the carfilzomib group and in 36% in the bortezomib group. The most frequent grade 3 or higher AEs were anemia (14% in the carfilzomib group vs. 10% in the bortezomib group), hypertension (9% vs. 3%), thrombocytopenia (8% vs. 9%), and pneumonia (7% vs. 8%). Grade 2 or higher PN was lower in the Kd group than in the BD group (6% vs. 32%).

Carfilzomib is currently approved with a twice-weekly schedule at a dose of 27 mg/m^2^ when administered in combination with lenalidomide and dexamethasone (KRd), or at 56 mg/m^2^ when given in combination with dexamethasone (Kd). The current twice-weekly schedule may prove burdensome for patients, especially if they are elderly and have limited access to hospital facilities. To improve the convenience of the carfilzomib schedule, preliminary studies tested higher doses of carfilzomib given in a once-weekly schedule. In the phase I/II CHAMPION-1 evaluating once-weekly carfilzomib dosing, the MTD was established as 70 mg/m^2^ in combination with dexamethasone [68]. Based on these results, the phase III study (A.R.R.O.W.) comparing the PFS of once-weekly carfilzomib with twice-weekly carfilzomib in patients with RRMM was conducted [62]. The once-weekly group received carfilzomib at 20 mg/m^2^ on day 1 during cycle 1, and then at 70 mg/m^2^ on days 8 and 15 of all cycles thereafter. The twice-weekly group received carfilzomib at 20 mg/m^2^ on days 1 and 2 during cycle 1, and then 27 mg/m^2^ thereafter. All patients received dexamethasone at a dose of 40 mg on days 1, 8, and 15 during all cycles and day 22 during cycles 1–9. The median PFS was significantly longer in the once-weekly group than in the twice-weekly group (11.2 months vs. 7.6 months, HR 0.69, *p* = 0.0029). The grade 3 or higher AEs was observed in 68% of patients in the once-weekly group and in 62% of them in the twice-weekly group. The incidence of anemia and thrombocytopenia were quite similar between both groups (18% and 7%, respectively). Pneumonia was observed in 10% of patients in the once-weekly group and in 7% of them on the twice-weekly group. Notably, the incidence of grade 3 or higher cardiac failure was similar between the once-weekly group and the twice-weekly group (4% vs. 3%). Based on these results, US FDA approved once-weekly carfilzomib in combination with dexamethasone for RRMM in 2018.

#### 4.1.2. Carfilzomib, Lenalidomide, and Dexamethasone (KRd)

Phase I and II studies showed the activity and tolerability of carfilzomib, lenalidomide, and dexamethasone (KRd) in relapsed myeloma patients. The PX-171-006 study assessed safety and tolerability of KRd regimen in 40 patients [69]. Carfilzomib was administered twice-weekly at doses of 15 mg/m^2^ to 27 mg/m^2^. Lenalidomide was administered in the first 21 days of a 28-day cycle, ranging from 10 mg a day to 25 mg a day. Dexamethasone was administered at a dose of 40 mg weekly. The MTD was not reached. Grade 3 or higher neutropenia was observed in 42.5% of the patients, anemia in 20%, thrombocytopenia in 32.5%, and lymphopenia in 27.5%, respectively. Grade 3 or higher fatigue was observed in 7.5% of the patients, diarrhea in 5.0%, and hyperglycemia in 22.5%, respectively. The ORR was 62.5% and the median DOR was 11.8 months for patients who achieved at least a PR. Median PFS was 10.2 months. The phase II dose-expansion study evaluating safety and efficacy was conducted in 52 patients treated at the maximum planned dose [70]. The ORR was 76.9% and median DOR of 22.1 months. Median PFS was 15.4 months and median time to response was 0.95 months. In bortezomib-refractory patients, the ORR was 69.2% and the median PFS was 15.4 months. In lenalidomide-refractory patients, the ORR was 69.9% and the median PFS was relatively short (7.9 months). Grade 3 or higher neutropenia was observed in 32.7% of the patients, anemia in 19.2%, thrombocytopenia in 19.2%, and lymphopenia in 48.1%, respectively. Fatigue and diarrhea were commonly observed (69.2% and 57.7%). Only one patient experienced grade 3 neuropathy. Cardiac AE of any grade was observed in 19.2% of patients, with three patients of grade 3 or higher.

Based on these findings, an ASPIRE trial comparing KRd with Rd in patients with relapsed MM was initiated [63]. The ASPIRE trial showed that the KRd group is superior in terms of PFS compared to the Rd group (26.3 vs. 17.6 months; HR 0.69, *p* = 0.0001). The ORR was 87.1% and 66.7% in the KRd and the Rd group, respectively (*p* < 0.001). The rate of a CR or better was 31.8% and 9.3%, and stringent CR rate was 14.1% and 4.3%. Adverse events of grade 3 or higher were reported in 83.7% and 80.7% of patients in the KRd and the Rd groups, respectively. The treatment discontinuation rate due to AE was similar between both groups (15.3% in the KRd and 17.7% in the Rd group). Recent report showed OS data from the ASPIRE trial. Median OS was 48.3 months for the KRd versus 40.4 months for the Rd group (HR 0.79, *p* = 0.0045). Selected grade 3 or higher AEs of interest included acute renal failure (3.8% in the KRd vs. 3.3% in the Rd group, respectively), cardiac failure (4.3% vs. 2.1%), ischemic heart failure (3.8% vs. 2.3%), and hypertension (6.4% vs. 2.3%). Overall, the KRd regimen is considered as one of the standards of care in patients with relapsed and refractory MM.

#### 4.1.3. Daratumumab, Carfilzomib, and Dexamethasone (DKd)

The number of patients treated with lenalidomide-based regimens as a front-line treatment has been recently increasing. As a result, the effective regimens for lenalidomide-refractory patients are needed. A phase Ib study evaluating daratumumab plus carfilzomib and dexamethasone (DKd) in patients with relapsed or refractory MM was reported [64]. In this study, eighty-five patients who did not receive carfilzomib and daratumumab were enrolled. Carfilzomib (20 mg/m^2^ on day 1 and thereafter 70 mg/m^2^) was given on days 1, 8, and 15 of each 28-day cycle. Dexamethasone was given at 40 mg once a week. Daratumumab was given as a single first dose of 16 mg/kg for ten patients or as a split first dose of 8 mg/kg on days 1 and 2 of cycle 2 for seventy-five patients. The ORR was 84% in the overall population compared with 79% in the lenalidomide-refractory population. The median PFS was not reached in the overall population compared with 25.7 months in the lenalidomide-refractory population. According to the cytogenetic risk group, the median PFS was not reached in the standard-risk group compared with 23 months in the high-risk group. Grade 3 or higher thrombocytopenia was observed in 31% of the patients, lymphopenia in 24%, anemia in 21%, and neutropenia in 21%, respectively. Infusion-related reactions were observed in 60% and 43% of single and split first-dose patients, respectively. These results suggested that DKd appears to be a well-tolerated and effective regimen in patients with relapsed or refractory MM, including refractory to lenalidomide. A randomized, open-label phase III study of DKd compared to Kd is ongoing.

## 5. Ixazomib

Ixazomib is an oral, selective and reversible PI, which inhibits the chymotrypsin-like activity of the β5 subunit of the 20S proteasome. Chemically, it is an N-capped dipeptidyl leucine boronic acid, which is rapidly hydrolyzed in water and converted into ixazomib. The proteasome dissociation half-life is shorter than bortezomib. Ixazomib treatment of MM cells induced caspase-mediated apoptosis, accompanied by induction of the unfolded protein response. In vitro synergy between ixazomib and lenalidomide was detected in two of four MM cell lines evaluated in viability assays, and two MM cell lines evaluated showed the additive effect of the combination [71].

### 5.1. Ixazomib-Based Therapy in Relapsed and/or Refractory MM

#### Ixazomib, Lenalidomide, and Dexamethasone (IRd)

A phase III, prospective and randomized, double-blind TOURMALINE-MM1 study of ixazomib plus lenalidomide and dexamethasone (IRd) versus placebo plus lenalidomide and dexamethasone (placebo-Rd) in refractory and/or relapsed MM previously treated with one to three lines was reported (Table 4) [65]. Ixazomib 4 mg was to be administered on days 1, 8, and 15 in combination with lenalidomide 25 mg on days 1–21 and dexamethasone 40 mg on days 1, 8, 15, and 22 in a 28-day cycle until progressive disease or unacceptable toxicity. The median PFS was 20.6 months in the IRd group compared with 14.7 months in the placebo-Rd group (HR 0.74, *p* = 0.01). Notably, IRd could overcome the negative impact of high-risk cytogenetics on PFS. The median PFS was 21.4 months in the high-risk group vs. 20.6 months in the standard-risk group. The ORR was significantly higher in the IRd group vs. in the placebo-Rd group (78.3% vs. 72%, *p* = 0.04), as well as the rates of CR plus very good PR (48% vs. 39%). At a median follow-up of 23 months, the median OS was not reached in either study group. The most frequently AEs were diarrhea (42% in the IRd group vs. 36% in the Rd group), constipation (34 vs. 25%), thrombocytopenia (28% vs. 14%), PN (25% vs. 18%), vomiting (22% vs. 11%) and back pain (21% vs. 16%). Rash was observed more frequently in the IRd group compared to the placebo-Rd group (36% vs. 23%). Thus, IRd regimen is one of the standards of care for patients with RRMM.

### 5.2. Ixazomib Maintenance Therapy

Recent studies have demonstrated that lenalidomide-maintenance therapy after ASCT could improve PFS and OS [72,73,74,75]. A meta-analysis showed that the discontinuation rate due to AEs was 29% in the lenalidomide maintenance group and 12% of the placebo or observation group [76]. On the other hand, bortezomib-maintenance therapy has also shown promising activity in ASCT setting [28,77,78]. However, there has been no phase III study demonstrating a survival benefit of PI-based maintenance therapy compared with placebo. In addition, the clinical utility of bortezomib as a maintenance treatment may be limited due to the need for parenteral administration and toxicity such as PN. Therefore, oral PI maintenance therapy including ixazomib suitable for long-term use without cumulative toxicity is needed [79].

#### Ixazomib Maintenance Following Autologous Stem Cell Transplantation

The phase III, double-blind, placebo-controlled TOURMALINE-MM3 study of ixazomib maintenance versus placebo in patients who underwent ASCT was conducted [80]. The study included 656 patients who achieved a PR to induction therapy followed by single ASCT. Patients were randomly assigned 3:2 to ixazomib or placebo group. Each agent was administered on days 1, 8, and 15 of each 28-day cycle. Ixazomib was administered at a dose of 3 mg during the first four cycles and was escalated to 4 mg after the fifth cycle if tolerated. With a median follow-up of 31 months, the median PFS (primary endpoint) was superior in the ixazomib group vs. the placebo group (26.5 months vs. 21.3 months, HR 0.72, *p* = 0.0023). In the subgroup analysis, PFS benefit of ixazomib maintenance was observed in patients who were aged 60 years or older, who had ISS disease stage III, and who had high-risk cytogenetics. In addition, depth of response improved during maintenance therapy in 46% of the patients in the ixazomib group and 32% of them in the placebo group. Serious AEs were observed in 27% of patients in the ixazomib group and 20% of those in the placebo group. The most common grade 3 or higher AEs were infections (15% in the ixazomib group vs. 8% in the placebo group), gastrointestinal disease (6% vs. 1%), neutropenia (5% vs. 3%) and thrombocytopenia (5% vs. <1%). The PN occurred in 19% of the patients in the ixazomib group versus 15% of them in the placebo group. Second malignancy was observed in 3% each. The ixazomib maintenance may become a new option in the ASCT setting, especially for patients who have high-risk features or are not tolerable for lenalidomide. However, a longer follow-up is needed to evaluate OS benefit.

## 6. Novel Inhibitors of Deubiqutinating Enzymes

Novel inhibitors targeting deubiquitinating enzymes (DUBs) or ubiquitin receptors upstream of 20S proteasome is under investigation. These agents may overcome PI-resistance. DUBs are antagonists of E3 ubiquitin ligases. These recognize ubiquitinated proteins and remove their ubiquitin tags by the protease activity [81]. Schwickart et al. demonstrated that DUB ubiquitin-specific-processing protease (Usp) 9x is highly expressed in MM cells and is involved in anti-apoptotic protein, Mcl-1 stabilization [82]. WP1130, a selective Usp9x inhibitor, leads to apoptosis and reduction of Mcl-1 expression in MM cells. Usp9x depletion induced upregulation of Usp24 which has shown to be involved in myeloma cell survival. Both Usp9x and 24 were expressed and activated in primary MM cells. EOAI3402153 is a novel inhibitor, which inhibits both Usps, and suppresses to cell survival and tumor growth in vivo [83]. In addition, a selective Usp7 inhibitor P5091 is being investigated in the preclinical study [84]. Higher expression of Usp7 has shown to be correlated with poor outcome in MM. On the other hand, novel inhibitors of 19S regulatory particle (RP) proteasome are also under investigation. Capzimin (CZM) inhibits the DUB Rpn11 of the 19S RP [85]. Before the entry and degradation of substrates in the 20S proteasome, the polyubiquitin chains of substrates need to be removed. Rpn11 is one of the subunits of lid of the 19S RP and removes the polyubiquitin chains. CZM has been shown to be active in several cancer cell lines, including bortezomib-resistant cells. At present, other two Rpn11 inihibitors, O-phenanthroline and thilutin, are also being investigated [86,87]. The further clinical studies of these inhibitors are awaited.

## 7. Future Perspectives

Triplet regimens for MM consisting of a PI, an IMiD or an alkylator, and dexamethasone have been evaluated and widely used in the clinical setting, as described above. To improve the survival of MM, achieving and maintaining a deeper response is important in patients who are both eligible and ineligible for transplantation. It appears that the quadruplet combination with novel agents is necessary to obtain the deeper response. The addition of monoclonal antibodies or alkylators to the traditional triplet induction regimens may be reasonable because of fewer additional side effects. However, the efficacy and safety of quadruplet regimens has, to date, not been fully elucidated. Thus, many clinical trials are ongoing. As described above, the results from part 1 of the CASSIOPEIA trial show the clinical benefit of the addition of daratumumab to VTD in transplant-eligible patients with newly diagnosed MM [30]. The phase III Myeloma XI trial randomized 1,056 transplant-eligible patients with newly diagnosed MM to either carfilzomib, cyclophosphamide, lenalidomide and dexamethasone (KCRD), cyclophosphamide, thalidomide and dexamethasone (CTD)/cyclophosphamide, lenalidomide, and dexamethasone (CRD) as an induction therapy before ASCT. Treatment with quadruplet regimen was associated with a significantly longer PFS than triplet therapy (HR 0.63, *p* < 0.001). The 3-year PFS rate was also significantly higher in the KCRD group than in the triplet group (64.5% vs. 50.3%, *p* < 0.0001). No additional toxicity was observed in the quadruplet regimen [88]. The randomized, open-label, phase II GRIFFIN study investigated the use of daratumumab in combination with VRd (D-VRd) in patients with newly diagnosed MM who were eligible for transplantation. This was compared to VRD-alone. The rate of sCR (primary endpoint) was significantly higher in the D-VRd group than in the VRd group (42% vs. 32%). In the D-VRd group, 59% of the patients were negative for MRD (10^−5^ sensitivity threshold), as compared with 24% of those in the VRd group. The safety profile of the D-VRd quadruplet was similar to that reported for the drugs when used separately [89]. These results indicate that the addition of daratumumab to traditional triplet induction regimens could improve efficacy. In transplant-ineligible patients, the quadruplet regimen has just been established [45]. As described above, D-VMP is effective and tolerable for this population.

In the clinical setting, lenalidomide maintenance or continuous therapy has been performed in both transplant-eligible and -ineligible patients with MM. Maintenance approaches incorporating PIs might favor a combination approach, especially in high-risk patients. A randomized phase II study comparing ixazomib alone with ixazomib plus lenalidomide as maintenance therapy is ongoing (NCT03733691). In addition, a randomized phase III study evaluating the impact on PFS when adding ixazomib to post-transplant maintenance therapy with lenalidomide and dexamethasone after ASCT in patients with newly diagnosed MM is also ongoing (NCT02406144). The phase II trial evaluating the efficacy and safety of bortezomib in combination with lenalidomide as maintenance therapy in high-risk patients with newly diagnosed MM who received VRd regimen as induction therapy is also ongoing (NCT03641456). These results will contribute to better understanding and decision making of the optimal maintenance therapy in MM.

## 8. Conclusions

PIs are an indispensable agent for increasing PFS and quality of life, and are achieving a deeper response, especially in subgroups of patients with poor prognosis. A number of clinical studies evaluating the safety and efficacy of new combination regimens involving PIs and the other novel agents are ongoing.

## Figures and Tables

**Table 1:** Chemical and pharmacological features of different proteasome inhibitors.

Agents	Active Moiety	Binding Kinetics	Therapeutic Targets	IC50 β5 (nM)	Half-Life (min)	Route of Administration
**Bortezomib (PS-341)**	Boronate	Reversible	β5 > β1	5.7	110	IV or SC
**Carfilzomib (PR-171)**	Epoxyketone	Irreversible	β5 > β2/β1	5	<30	IV
**Ixazomib (MLN9708)**	Boronate	Reversible	β5 > β1	5.9	18	Oral
**Marizomib (NPI-0052)**	β-Lactone	Irreversible	β5 > β2 > β1	9.1	10-15	IV or SC
**Oprozomib (ONX0912)**	Epoxyketone	Irreversible	β5	6-12	30-90	Oral
**Delanzomib (CEP-18770)**	Boronate	Reversible	β5 > β1	5.6	62 (hrs)	Oral

Ⅳ: intravenous, SC: subcutaneous, IC50: half maximal inhibitory concentration, adapted from Moreau P. et al. [6] and Teicher B.A. et al. [7].

**Table 2:** Selected bortezomib-containing regimens for upfront treatment of transplant-eligible MM.

Trials	Phase	Patients, *n*	ORR (CR)	Median PFS (Months)	Median OS (Months)
IFM2005-01 [23] (VD vs. VAD)	Ⅲ	482	78.5% vs. 62.8% (≥nCR:14.8% vs. 6.4%)	36.0 vs. 20.7	81.4 vs. 77.4
DSMM-XI [24] (VCD)	Ⅱ	414	85.4% (7.4%)	35.3	NR
GIMEMA [25] (VTD vs. TD)	Ⅲ	480	93% vs. 79% (≥nCR: 31% vs. 11%)	68% vs. 56% at 3-yr	86% vs. 84% at 3-yr
GEM05-MENOS65 [26] (VTD vs. TD vs. CTx + V)	Ⅲ	386	85% vs. 64% vs. 75% (≥nCR:35% vs. 14% vs. 21%)	56.2 vs. 28.2 vs. 35.5	(not reported)
IFM2013-04 [27] (VTD vs. VCD)	Ⅲ	340	>VGPR:66.3% vs. 56.2% (13% vs. 8.9%)	(not evaluated)	(not evaluated)
HOVON-65/GMMG-HD4 [28] (PAD vs. VAD)	Ⅲ	744	78% vs. 77% (≥nCR: 31% vs. 15%)	36 vs. 27	78% vs. 70% at 3-yr
IFM2009 [29] (VRD ± ASCT)	Ⅲ	700	99% vs. 97% (59% vs. 48%)	50 vs. 36	81% vs. 82% at 4-yr
CASSIOPEIA [30] (D-VTD vs. VTD)	Ⅲ	1085	92.6% vs. 89.9% (sCR: 29% vs. 20%)	NR vs. NR	(not reported)

VD, bortezomib + dexamethasone; VCD, bortezomib + cyclophosphamide + dexamethasone; TD, thalidomide + dexamethasone; VTD, TD + Bortezomib; CTx + V, chemothertapy + bortezomib; PAD, doxorubicin + bortezomib + dexamethasone; VAD, vincristine + doxorubicin + dexamtheasone; VRD, bortezomib + lenalidomide + dexamethasone; ASCT, autologous stem-cell transplantation; D-VTD, daratumumab + VTD; ORR, overall response rate; CR, complete response; nCR, near CR; sCR, stringent CR: VGPR, very good partial response; NR, not reached; PFS, progression-free survival; OS, overall survival.

**Table 3:** Selected bortezomib-containing regimens for upfront treatment of transplant-ineligible MM.

Trials	Phase	Patients, *n*	ORR (CR)	Median PFS (Months)	Median OS (Months)
VISTA [44] (VMP vs. MP)	Ⅲ	682	71% vs. 35% (30% vs. 4%)	TTP 24.0 vs. 16.6	56.4 vs. 43.1
ALCYONE [45] (D-VMP vs. VMP)	Ⅲ	706	90.9% vs. 73.9% (42.6% vs. 24.4%)	36.4 vs. 18.1	NR vs. NR
SWOG-S0777 [46] (VRd vs. Rd)	Ⅲ	525	82% vs. 72% (16% vs. 8%)	43 vs. 30	75 vs. 64
VRd Lite [47]	Ⅱ	50	86% (≥VGPR 66%)	35.1	NR

MP, melphalan + prednisone; VMP, bortezomib + MP; D-VMP, daratumumab + VMP; Rd, lenalidomide + dexamethasone; VRD, bortezomib + Rd; ORR, overall response rate; CR, complete response; VGPR, very good partial response; PFS, progression-free survival; TTP, time to progression; OS, overall survival; NR, not reached.

**Table 4:** Selected carfilzomib or ixazomib-containing regimens for relapsed and/or refractory MM.

Trials	Phase	Patients, *n*	ORR (CR)	Median PFS (Months)	Median OS (Months)
ENDEAVOR [61] (Kd vs. Vd)	Ⅲ	929	77% vs. 63%	18.7 vs. 9.4	47.6 vs. 40.0
A.R.R.O.W. [62] (weekly Kd vs. twice-weekly Kd)	Ⅲ	478	62.9% vs. 40.8% (7.1% vs. 1.7%)	11.2 vs. 7.6	(not reported)
ASPIRE [63] (KRd vs. Rd)	Ⅲ	525	82% vs. 72% (16% vs. 8%)	26.3 vs. 17.6	48.3 vs. 40.4
DKd [64]	Ib	85	84% (33%)	NR (74% and 66% at 12-and 18- months)	NR (82% at 12-months)
TOURMALINE-MM1 [65] (IRd vs. Rd)	Ⅲ	722	78% vs. 72% (12% vs. 7%)	20.6 vs. 14.7	NR vs. NR

Kd, carfilzomib + dexamethasone; Vd, bortezomib + dexamethasone; Rd, lenalidomide + dexamethasone; KRD, carfilzomib + Rd; DKd, daratumumab + Kd; IRd, ixazomib + Rd; ORR, overall response rate; CR, complete response; PFS, progression-free survival; OS, overall survival; NR, not reached.
